# The impact of economic and social factors on the prevalence of hepatitis B in Turkey

**DOI:** 10.1186/s12889-018-5575-6

**Published:** 2018-05-22

**Authors:** Selma Tosun, Olgu Aygün, Hülya Özkan Özdemir, Elif Korkmaz, Durmuş Özdemir

**Affiliations:** 1 Department of Clinical Microbiology and Infectious Diseases, University of Health Sciences, Bozyaka Education and Research Hospital, İzmir, Turkey; 2 Number 2 General Practioner Center, Bozyaka Education and Research Hospital, İzmir, Turkey; 3 Department of Economics, Yaşar University, Üniversite Caddesi No: 37-39, 35040 Bornova, İzmir, Turkey

**Keywords:** Hepatitis B virus, Economic and social factors, Prevalence, Turkey

## Abstract

**Background:**

Viral Hepatitis is one of the major global health problems, affecting millions of people every year. Limited information is available on the impact of social and economic factors on the prevalence of Hepatitis B virus (HBV) in Turkey. This study, contrary to other studies in the literature, was undertaken with the aim of examining the Majority of the excluded data come from the volunteers.

**Methods:**

There are medical and the social-economic factors affecting the prevalence of HBV. This research, while taking medical factors as control variables, clarify the social and economic factors affecting the prevalence of HBV by utilising clinical data with the use of the Binary Probit Model (BPM). The BPM estimation is a powerful tool to determine not only the factors but explain also the exact impacts of each factor.

**Results:**

The estimations of the BPM shows that economic and social variables such as age, gender, migration, education, awareness, social welfare, occupation are very important factors for determining HBV prevalence. Compared to the youngest population, the 46 to 66+ age group has a higher prevalence of HBV. The male respondents were 5% more likely to develop HBV compared to females. When region-specific differences are taken into account, migrating from the poorest parts of the country such as the eastern and south-eastern regions of Turkey are approximately 16% more likely to be infected. The welfare indicators such as a higher number of rooms in the respondent’s house or flat decreases the probability of having HBV and, relatively higher income groups are less likely to develop HBV compared to labourers. The Self-employed/Business owner/Public sector worker category are approximately 10% less likely to develop HBV. When people are aware of the methods of prevention of HBV, they are 6% less likely to be infected. Previous HBV infection history increases the probability of having HBV again B by 17%.

**Conclusions:**

These findings strongly suggest that the impact of social and economic factors on the prevalence of HBV is vital. Any improvements in these factors are likely to reduce prevalence of HBV.

## Background

Hepatitis B Virus (HBV) is one of the major global health problems, affecting millions of people every year and causing disability and death. An estimated 2 billion people are infected with Hepatitis B, with more than 240 million being chronically infected. These Hepatitis B-related infections result in 500,000 to 700,000 deaths per year [1–3]. World Health Assembly endorsed a global action plan called *Global Health Sector Strategy on Viral Hepatitis 2016–2021* (GHSS) to tackle viral hepatitis infection with a particular attention to Hepatitis B and C. The GHSS aims to eliminate viral hepatitis as a public health threat by 2030 aligned with the 2030 Agenda for Sustainable Development. The strategy set targets for the year 2020 and 2030 for all countries to achieve a set of ambitious goals which are relevant to our study. They are, the reduction for new cases of chronic hepatitis B and C (%30 by 2020 and 90% by 2030), reduction for mortality from viral hepatitis (%10 by 2020 and %65 by 2030). Moreover, childhood vaccine coverage for HBV (%90 by 2020 and 2030) and prevention of HBV mother-to-child transmission (%50 by 2020 and %90 by 2030). WHO also set goals for diagnosis and treatment of HBV. Their strategy aims to increase number of people with chronic hepatitis infections diagnosed from less than %5 (in 2015) to 30% by 2020 and %90 by 2030 while 80% of eligible persons treated by 2030.

HBV is highly infectious and, according to the WHO, there are a number of factors affecting the prevalence of HBV. Turkey has been placed by the WHO into the intermediate zone of prevalence for HBV; however the mass migration affect from East of the country and Syria are unknown. Epidemiological studies reveal that Hepatitis B surface antigen (HBsAg) positivity in Turkey has been reported between 4 and 5%. Moreover, there are region-specific differences in the prevalence of Hepatitis B in Turkey [1, 4, 5]. However, The Turkish Ministry of Health stated that the incidence of hepatitis B infection decreases over time. In 2002 the number of incidence of Hepatitis B infection was 8.26 per 100,000 people in Turkey. This number slightly decreased in 2008 to 8.18 and a sharp decline in 2010 which was 4.26 per 100,000 people [6]. Government commitment to fight with Hepatitis B infection plays a crucial role on the downward trend of infection. Universal Hepatitis B vaccination policies were implemented in Turkey and requires a routine vaccination program. All new-born were vaccinated at birth since 1998. Besides new-borns, Hepatitis B Control Program launched by the Ministry of Health in 2008 covers vaccination of adolescents and adults in the risk groups [7]. Lastly, according to the latest data, Turkey is projected to meet the 2030 target of ≤0.1% HBsAg prevalence among 5 year olds [8].

There are a few studies attempting to show the link between some of the social and economic factors such as migration and the prevalence of a disease in the literature [9–15]. They carried out a regression analysis to identify risk factors for infection and coinfection for HBV,HCV, and HIV and examined the predictors of under immunization among the 2006 birth cohort in the state’s immunization information system, including individual demographic and socioeconomic status (SES) data. They conducted multilevel logistic regression data and concluded that the efforts focused on vaccinating infants born in low SES circumstances can minimize disparities. A study conducted Cochran-Mantel-Haenszel analysis and argued that the intervention effect on screening outcomes remained statistically significant after adjustment for demographic and health care access variables including income, having health insurance and having a regular health provider [16]. Yet there are very few published studies specifically focusing on the socioeconomic aspect of the prevalence of HBV and there are no study, which covers all of the socio economic factors. Therefore, the objective of our paper was to explain the role of all possible social and economic factors, such as the age, gender, migration, occupation, social status, employment, education, awareness, and history of HBV, on hepatitis B prevalence. The clarification of social and economic factors are a serious issue, which help to reduce the diseases caused by HBV and any improvements in these factors likely to increase the health conditions of the millions of people who are in the risk category.

## Methods

### The statistical method and the model

Because the dependent variable is binary in nature, binary response models that directly describe the response probabilities of the dependent variable are appropriate. In literature, logit and probit regression models have often been used to assess risk factors for various diseases [16–20]. Although these models produce different parameter estimates, they end up with almost the same standardised impacts of independent variables [21]. Following these, this study employs a binary probit regression model. The model calculates the maximum estimates of regression parameters and the natural response rate for the discrete event data. Probit model can be derived by an underlying latent variable. Within this framework, y* is the latent variable, which indicates the likelihood of having Hepatitis B. The baseline model is as follows:

$$ {y}_i\ast ={\beta}_0+\beta {x}_i+e $$

where *x* is the vector of explanatory variables (i.e., socio-economic and demographic factors), *β* is the vector of parameters and *e* is the error term that is normally distributed and independent from *x*. Because the latent variable is unobservable in practice, an indicator variable should be attained which is considered as *HepB* in this study. *HepB* takes a value of one if the patient is Hepatitis B positive and zero otherwise. Formally:

$$ {HepB}_i=\left\{\begin{array}{c}1\  if\ {y}_i\ast >0\\ {}0\  if\ {y}_i\ast \le 0\end{array}\right. $$

Response probability of *HepB* is given as follows:

$$ \left\{\begin{array}{c}P\left({HepB}_i=1\right)=P\left({y}_i\ast >0\right)=G\left({\beta}_0+\beta {x}_i\right)\\ {}P\left({HepB}_i=0\right)=P\left({y}_i\ast \le 0\right)=G\left({\beta}_0+\beta {x}_i\right)\end{array}\right. $$

where G is the standard normal cumulative distribution function.

### Data and variables

The data sample was conducted İzmir, Turkey, where families belonged to socio-economically low and middle class levels. Selected region represents the general socio-economic structure of the Turkish population. Volunteers registered to family medicine centre of the district chosen to be part of the survey and were selected randomly. This is a cross sectional study of 1112 volunteers, of which 924 were used in this analysis. To be eligible to join the study, volunteers should be older than 16 years old. A person who is younger than 16 and who is vaccinated against Hepatitis B is excluded from the data set because compulsory vaccination was started 19 years ago in Turkey. One hundred and eighty-eight volunteers were eliminated from the data set, either, because of incomplete questionnaires as they failed to complete the questionnaire forms/ incomplete clinical data (38 volunteer) or they were vaccinated against Hepatitis B (150 volunteer). Of these 924 volunteers, 312 were male and 612 were female. The median age is 42 and ranges 16–89 years. Regional differences are also examined while 118 of the volunteers are migrated from eastern Turkey. The data were presented as frequencies and percentages for categorical variables and the mean and standard deviation for continuous variables listed in Table 1 below. Medical tests determined that 213 volunteers were infected with the Hepatitis B virus. The questionnaire was designed by the researchers to assess differences in the socio-economic and demographic characteristics of patients, as well as their knowledge and awareness of Hepatitis B.

**Table 1:** Baseline characteristics

	Number	Percent		Number	Percent	
*AGE*			*AWAREP*			
*16–25*	109	12.1	Yes	271	29.88	
26–35	196	21.7	No	13	1.43	
36–45	237	26.2	Don’t Know	623	68.69	
46–55	200	22.1	AWAREPC			
56–65	101	11.2	Yes	68	7.71	
66+	61	6.7	No	112	12.7	
GENDER			Don’t Know	702	79.59	
Male	312	33.8	VIRALHEP			
Female	612	66.2	Yes	647	70.94	
*OCCUPATION*			No	213	23.36	
*Labourer*	192	21.4	Don’t Know	52	5.7	
Unemployed	247	27.5	PATIENT			
Retired	131	14.6	I hear	397	43.44	
Self-employed /B.O/P.S.Worker	327	36.5	I don’t hear	404	44.2	
INTERNET			Don’t Know	113	12.36	
Yes	559	63	HISTofHBV			
No	329	37.1	Yes	102	11.26	
CELLPHONE			No	654	72.19	
Yes	878	97	Don’t Know	150	16.56	
No	27	2.98		Obs	Mean	Std. Dev.
MIGRANT			ROOM	905	3.3	0.75
Yes	382	57.5	PEOPLE	898	3.53	1.22
No	517	42.5				
MIG_EAST						
Yes	118	87.2				
No	806	12.8				

Following ethical committee approval and written consent from volunteers, face-to-face interviews were conducted and questionnaires were correctly completed. An enzyme immunoassay (EIA) method was used to detect HBV (HBsAg, AntiHBcIgG, AntiHBS) through 7 cm^3^ blood samples. AntiHBcIgG is considered as a specific marker of Hepatitis B infection. The volunteer is considered as chronically infected if both HBsAg and AntiHBcIgG are positive. If both AntiHBcIgG and AntiHBS are positive than he/she is considered as naturally infected and immune. If all test results are negative then this implies that the volunteer is not infected with Hepatitis B. If the test of AntiHBs is positive, then the patient is considered as vaccinated against HBV.

As shown in Table 2, *HepB*_*i*_ is a dependent variable and indicates whether an individual was infected with Hepatitis B virus or not. The binary variable takes a value of one if the individual has Hepatitis B and zero otherwise.

**Table 2:** The list of variable definitions used in the empirical analysis

Dependent Variable	Definition
${HepB}_{\dot{I}}$	1 = if individual is infected with Hepatitis B virus; 0 = otherwise
Independent Variables	Definition
*AGE*_*i*_	Age of individuals (years)
*GENDER*_*i*_	1 = male; 0 = female
*OCCUP*_*i*_	Labourer = 0
	Unemployed/Does not work = 1
	Retired = 2
	Self-employed/Business owner/Public sector worker = 3
*ROOM*_*i*_	Number of rooms in the house
*PEOPLE*_*i*_	Number of people in the house
*INTERNET*_*i*_	1 = if individual use internet; 0 = otherwise
*CELLPHONE*_*i*_	1 = if individual has a cell phone; 0 = otherwise
*MIG*_*i*_	1 = if individual is a migrant; 0 = otherwise
*MIG*_*EAST*_*i*_	1 = if individual is migrated from south-eastern and eastern Anatolia;
	0 = otherwise
*AWAREP*_*i*_	Hepatitis B is preventable(P):
	Don’t know = 0
	Yes = 1
	No = 2
*AWAREPC*_*i*_	Hepatitis B is transmitted by physical contact (PC):
	Don’t know = 0
	Yes = 1
	No = 2
*VIRALHEP*_*i*_	Have you ever heard of viral Hepatitis:
	Don’t know = 0
	Yes = 1
	No = 2
*PATIENT*_*i*_	Have you ever heard someone diagnosed with Hepatitis B:
	Don’t know = 0
	Yes = 1
	No = 2
*HIST*_*i*_	Have you ever had HBV before:
	Don’t know = 0
	Yes = 1
	No = 2

The set of explanatory variables vector covers socio-economic and demographic characteristics of individuals and their knowledge about the disease. *AGE*_*i*_ is measured in years and divided into six subgroups in order to explore the significant age group among others. Age groups were 18–25, 26–35, 36–45, 46–55, 56–65 and above 66 years. *GENDER*_*i*_ is a dummy variable that takes a value of one if the patient is male and zero if the patient is female. This variable helps to see whether there are significant gender differences in the propensity for having Hepatitis B.

*OCCUP*_*i*_ is a categorical variable and represents the householder’s occupational status. The base category is being a labourer. This variable takes a value of one if the householder is unemployed/not working and takes a value of two if the householder is retired. The last category is Self-employed/Business owner/Public sector worker. This variable was used as a proxy for family income under the idea that this covariate is one of the major predictors of risk status. The *ROOM*_*i*_ variable was also created to indicate economic welfare and measured by the number of room in the respondent’s house.

Besides reflecting the individual’s economic status, this variable also indicate shared risky conditions in the house because physical proximity to an infected patient is one of the possible causes of transmission. Percutaneous or mucosal exposures to blood or infectious body fluids create a high level of risk for people sharing a house with someone who is chronically infected [22]. The *PEOPLE*_*i*_ variable shows the number of people living in the house. *INTERNET*_*i*_ and *CELLPHONE*_*i*_ are dummy variables and take a value of one if the individual use internet/has cell phone and zero otherwise. These variables are also reflect the economic status of an individual in Turkey. Moreover, access to the internet is closely related to the ability to receive information, which is crucial to reach information about the Hepatitis B.

Migration dummy *MIG*_*i*_ is a variable takes a value of one if the individual is a migrant and zero otherwise. According to the literature, there are large differences in the prevalence of Hepatitis B among the different regions of Turkey. Differences arise from different socio-economic statuses, lifestyles, infrastructure and access to health services in various regions. Because of the poor living and health conditions, people in the eastern and south-eastern regions of Turkey are more likely to have Hepatitis B [23, 24]. In order to capture idiosyncratic characteristics of regions, the *MIG*_*EAST*_*i*_ dummy variable was created and takes a value of one if the individual migrated from eastern or south-eastern Anatolia and zero otherwise.

Hepatitis B immunisation has been available since 1982 and was recommended by the World Health Organisation (WHO) in 1992; Hepatitis B vaccinations are now included in children’s national immunisation programmes [25]. In Turkey, universal Hepatitis B vaccination policies were implemented in 1998 and have recently celebrated their 19th anniversary. Because the Hepatitis B vaccine is an effective way to prevent infection, knowledge about the methods of prevention plays a crucial role in the propensity of developing Hepatitis B. Accordingly, awareness of the prevention of Hepatitis B is measured by several questions in the survey study.

*AWAREP*_*i*_ is a categorical variable that takes a value of 1 if the respondent aware of the fact that Hepatitis B is preventable and takes a value of two if he/she believes that it is not preventable. The base category represents the last category of patients who have no knowledge of the methods of prevention of Hepatitis B. *AWAREPC*_*i*_ was conducted in a same manner in order to understand the patients’ knowledge of the mode of transmission. The variable takes a value of one if the respondent is aware of the fact that physical contact transmits Hepatitis B and a value of two if he/she believes the opposite. The base category represents patients who have no knowledge about the transmission of Hepatitis B.

*VIRALHEP*_*i*_takes a value of one if the respondent ever heard of viral hepatitis and a value of two if he/she has not heard. *PATIENT*_*i*_ takes a value of one if the respondent ever heard someone diagnosed with Hepatitis B and a value of two if he/she has not heard. The base category represents no knowledge in both variables.

The last variable *HISTofHBV*_*i*_ is also a categorical variable to measure if the respondent has ever had Hepatitis before. The variable takes a value of one if the respondent declares that they have never have Hepatitis before and takes a value of two if they have. The base category represents respondents who have no knowledge of their disease history.

## Results

This section presents the empirical results obtained from the estimation of binary probit model in order to explain factors affecting the prevalence of Hepatitis B. We use backward selection method to build the econometric model. This method uses Wald test for individual parameters. Cut-off *p* value is selected as 0.2 and independent variables with a *p*-value higher than this criterion are removed from the model [26, 27]. Final model is presented in the table below. When using a probit model, marginal effects should be obtained in order to have the estimated effects of the explanatory variables on the probability of a positive outcome. In line with this objective, the marginal effects of the factors on the probability of developing Hepatitis B are presented in the last column.

Table 3 shows the regression results of the pooled data. The first column of Table 3 represents the probit results of a baseline model, second column represents the logit results and the last column shows the marginal effects at the mean. The marginal coefficients explain by how much a unit increases (decreases) when the regressors increase (decrease) the probability of a positive outcome. Overall, the probit model works well and all variables in the specification have the expected outcome. In order to see how the fitted model reflects the real data, this paper employs the widely used Hosmer and Lemeshow goodness-of-fit test. Finding a *p*-value at the > 0.05 significance level (0.59) reveals that the model is fitting well. To compare the Logit and Probit models as a measure of the goodness of fit, the Akaike Information Criterion (AIC) is employed. From the considered models, the probit model has a lower AIC than the logit model, which means that probit is appropriate (*AIC*_*probit*_ = 766.03, *AIC*_*logit*_ = 767.15).

**Table 3:** Binary probit estimation results

	Probit	Logit	Robust	
HepB	Coef.	Coef.	Std. Err.	Marginal Coef.
*AGE*				
26–35	0.6182917^c^	1.1523^b^	0.2404455	0.0957449
36–45	0.7521858^c^	1.4164^c^	0.2339047	0.1262061
46–55	1.240986^c^	2.2215^c^	0.2385664	0.2668395
56–65	1.607314^c^	2.8608^c^	0.2664337	0.3951672
66+	1.297243^c^	2.365^c^	0.3070495	0.2855946
GENDER	0.196071^a^	0.3241^a^	0.112948	0.051475
MIG	0.3735635^c^	0.6241^c^	0.1197581	0.0966658
MIG_EAST	0.5586533^c^	0.9339^c^	0.1542164	0.1614965
ROOM	−0.1594673^b^	−0.2585^b^	0.076095	−0.0409056
*OCCUPATION*				
Unemployed	−0.1855878	−0.2969	0.1396187	−0.0499817
Retired	−0.2726053^a^	−0.4819^a^	0.1498429	−0.0715018
Self-employed/Business owner/Public sector worker	−0.3833353^b^	−0.7305^b^	0.184846	−0.0970206
*AWAREP*				
Yes	−0.2377457^a^	−0.4011^a^	0.1321559	−0.0590701
No	−0.024068	−0.0105	0.3847716	−0.0064049
AWAREPC				
Yes	0.1700024	0.2591	0.2026798	0.0445584
No	0.2951061^a^	0.5498^a^	0.1665921	0.080163
HISTofHBV				
Yes	0.555006^c^	−0.3578^c^	0.196325	0.1749221
No	−0.2128235	0.9572	0.1497063	−0.0551039
Cons	−1.223582		0.3656654	
Observations	793	793		
Pseudo R2	0.1478	0.1478		
Log-Likelihood	− 364.01669	−364.0167		

^a^Note: Statistically significant z-statistics at the 10, 5 and 1% levels stated as ^a^, ^b^ and ^c^ respectively

From a demographic point of view, the regression results show that *AGE* is statistically significant at the 1% significance level in all subgroups. Compared to the youngest population, the 46 to 66+ age group has a higher prevalence of Hepatitis B. It is important to highlight that the 56–65 age group has the highest negative marginal impact and is approximately 40% more likely to develop Hepatitis B, holding all other regressors constant. With the help of the universal Hepatitis B vaccination policies that were implemented in 1998, prevalence in the youngest population is relatively low, as expected. The results support the idea that Hepatitis B prevention strategies should be developed for elderly people born before the era of universal vaccinations.

The demographic variable *GENDER* is statistically significant as well. As the table shows above, male respondents were 5% more likely to develop Hepatitis B compared to females, holding all other regressors constant. Parallel to previous studies, the signs of the variables match expectations [11, 28, 29].

Migration related variables are also statistically significant at the 1% significance level. The results reveal that emigrated people are less likely to have Hepatitis B. However, the *MIG_EAST* variable points out an important consideration. When region-specific differences are taken into account, migrating from the eastern and south-eastern regions of Turkey increases the probability of having Hepatitis B. The results support that eastern and south-eastern migrants are approximately 16% more likely to be infected; the low socio-economic status, relatively low level of income and the low standard of living conditions in these two regions may contribute to the increased risk of developing the disease. These idiosyncratic differences in regions must be taken into account when implementing strategies for Hepatitis B prevention.

On the side of welfare indicators, *ROOM* is significant at the 1% significance level and negatively correlated with the regressand. A higher number of rooms in the respondent’s house or flat decreases the probability of having Hepatitis B. Another indicator of wealth is the occupational status of the householder named as the *OCCUP*.

When examining the marginal impact of the respondent’s occupational status, relatively higher income groups are less likely to develop Hepatitis B compared to labourers. Specifically, respondents in the Self-employed/Business owner/Public sector worker category are approximately 10% less likely to develop Hepatitis B. To sum up, the household income and economic wealth of individuals plays a crucial role because of their direct effect on living conditions, access to health services and medical care.

Regression results indicate the significance of the respondent’s knowledge and awareness of the disease on the probability of having Hepatitis B. As seen from the table above, respondents aware of the methods of prevention of Hepatitis B were approximately 6% less likely to be infected with the disease. Parallel to previous studies, a lack of information about the transmission of the disease increases the risk of having Hepatitis B [15, 20].

Respondents who had a lack of information about the transmission of the disease were 8% more likely to develop Hepatitis B. Awareness and the knowledge about the prevention and transmission of Hepatitis B among respondents are crucial factors for promoting the testing and for identification of those infected.

The last explanatory variable, *HISTofHBV,* also had a significant and positive marginal effect on the probability of Hepatitis B infection; previous Hepatitis infection increases the probability of having Hepatitis B by 17%, holding all other regressors constant. The underlying reasons might be a triggering effect on the spread of the disease, namely lifestyle, economic conditions and a lack of information on the methods of treatment and prevention of the disease.

## Discussion

The results of this study strongly suggest that the prevalence of HBV infection is associated with socioeconomic parameters. These parameters include age, sex, social welfare level, education level, awareness, migration and HBV history; not only have we identified the factors affecting HBV prevalence, we also know the impact of each variable on the prevalence of the disease.

Although %10 significance level weakens the strength of gender effect, analysis by gender reveals that, the male respondents were 5% more likely to develop HBV compared to females; this result comparable to other reports [10, 30], and the plausible explanation probably due to the higher exposure to occupational HBV risk factors in men. HBV and migration is important, as in our sample shows that immigrating people from the poorest parts of the country are approximately 16% more likely to be infected. The region-specific prevalence of HBV studies support our study [1].

There is no doubt that Impact of better education on health is exist. There is a need to educate masses on HBV [31],. According to the relevant literature, there are two channels for this. First, higher income allows people to purchase goods that improve health, for example, health insurance. In addition, higher income increases steady-state consumption, and thus raises the utility of living to an older age [32].

Our welfare indicators such as the number of bedrooms in the house or the occupational status of households are statistically significant at the 1% significance level and negatively correlated with the probability of having HBV. Wealthier they are, less likely to be infected.

Although, %10 confidence interval weakens our results on the awareness issue, there is a large literature confirm our current results. We focus here on the impact of awareness, and the respondents aware of the methods of prevention of Hepatitis B were approximately 6% less likely to be infected with the disease. Parallel to previous studies, a lack of information about the transmission of the disease increases the risk of having HBV [15, 20, 33, 34].

On the age related prevalence of HBV, the regression results show that AGE is statistically significant at the 1% significance level in all subgroups. As the population aged there is a higher prevalence of Hepatitis B, except the highest age group 65+. The relatively low prevalence rate of the highest age group can be explained with the fact that they are no longer in the high risky environment such as being less mobile and having less contact with the risk factors. Our observation on the progressive decrease, after 56 age, is accepted in the literature as after 50 [10, 35].

## Conclusion

There are medical and the social-economic factors affecting the prevalence of HBV. This research, while taking medical factors as control variables, clarify the social and economic factors affecting the prevalence of HBV by utilising clinical data with the use of the Binary Probit Model (BPM).

With the help of the universal Hepatitis B vaccination policies that were implemented in 1998, prevalence in the youngest population is relatively low, as expected. The results support the idea that Hepatitis B prevention strategies should be developed for elderly people born before the era of universal vaccinations. [36]. The most crucial policy advice we have is to increase compulsory vaccination programmes for all age groups. Universal vaccination seems justifiable on the basis of economic evaluation. Increasing the educational level of society as a whole and increasing society’s awareness and knowledge of the disease society, as well as introducing special programmes for migrants from the east (i.e., screening and vaccinations), would likely decrease the spread of HBV.

## Abbreviations

AGE: Age group of patients
AWAREP: HBV awareness variable
BPM: Binary probit model
EIA: Enzyme immunoassay
GENDER: Dummy variable that takes a value of one if the patient is male and zero otherwise
HBV: Hepatitis B virus
HCV: Hepatitis C virus
HISTofHBV: Variable for the HBV history
HIV: Human immunodeficiency virus
MIG: Migration variable
MIG_EAST: Mıgration from the east and the south east of the country
OCCUP: Categorical variable and represents the households occupational status
ROOM: The number of rooms in the house as a welfare indicator
SES: Socioeconomic status
WHO: World health organization
